# The economic effect of financial compensation in China’s healthcare system: comprehensive insights regarding supply and demand factors

**DOI:** 10.1186/s13561-024-00496-5

**Published:** 2024-03-16

**Authors:** Yi Guo, Xuezhi Hong, Dongmei Li, Qiannan An, Wenwen Fan, Minghe Yang, Luyang Xiao

**Affiliations:** Beijing University of Chinese Medicine, No. 11 North Third Ring East Road, Beijing, China

**Keywords:** Financial compensation, Healthcare system, Supply and demand, Novel medical reform, Regional heterogeneity.

## Abstract

**Objectives:**

We aim to analyse the effects of government subsidies on residents’ health and healthcare expenditure from the perspectives of supply and demand.

**Data and methods:**

According to the regional division adopted in the data query system of the National Bureau of Statistics, this study divides 31 provinces and cities into three regions: eastern, central, and western. The data used are from public databases, such as the “China Statistical Yearbook,” “China Health Statistical Yearbook,” and “Government Final Account Report”. In this study, mathematical model derivation is used to construct a fixed effects model, and an empirical study based on cross-sectional data and general linear regression is conducted. To prevent endogeneity issues, this study introduces instrumental variables and uses 2SLS regression to further analyse the output results.

**Results:**

For every 1% increase in supplementary funding on the supply side, the perinatal mortality rate decreases by 1.765%, while for every 1% increase in financial compensation on the demand side, per capita outpatient expenses increase by 0.225% and per capita hospitalization expenses increase by 0.196%. Regarding medical resources, for every 1% increase in the number of beds per 1,000 people, per capita hospitalization expenses decrease by 0.099%. In the central and eastern regions, where economic levels are higher, supply-side government funding is more effective than demand-side funding. In contrast, demand-side funding is more effective in the western region.

**Conclusions:**

The roles of multiple influencing factors and significant regional heterogeneity are clarified. Increasing financial compensation to providers positively impacts perinatal mortality but leads to higher per capita outpatient and hospital expenditures. Finally, this study provides targeted policy recommendations and solid theoretical support for policymakers.

**Supplementary Information:**

The online version contains supplementary material available at 10.1186/s13561-024-00496-5.

## Introduction

Limited healthcare financial scale is a significant challenge to government expenditure in China and concerns important livelihood issues [1] [2]. Inadequate allocation of public expenditure can lead to problems such as unequal distribution of healthcare resources, inefficient healthcare systems, and regional disparities in healthcare standards [3]. Reforming financial compensation for the healthcare system can support the effective and reasonable allocation of funds [4].

China’s financial compensation system has undergone several adjustments, and its development can be divided roughly into several stages [5]. The first stage is the period from the founding of New China to 1978, during which the government did not provide financial compensation and assumed the responsibility of only a “supplementary provider” [6]. The second stage was from 1978 to 1996, during which time the government still assumed responsibility of the supply side, but its input declined significantly [7]. The third stage covers the period from 1997 to 2008, during which the government gradually assumed the responsibility of “supplementary demand,” and its commitment to compensating suppliers began to erode [8]. The government is still seeking to determine its role in the healthcare system.

The UK and French governments are prime examples of governments that provide “one-sided” healthcare subsidies, injecting funds into only the supply side or the demand side, and they are facing financial crises [9] [10]. However, providers and patients are two main components of the healthcare compensation system in addition to the government [11]. Moreover, supplementary suppliers and demanders have become essential factors and research priorities in China’s healthcare policy [12]. The German government contributes to both the “supply side” and the “demand side,” covering health insurance costs for families without a source of income and remaining responsible for the financial input of public hospitals [13]. In the United States, the federal or local government also assumes responsibility for healthcare providers’ financial reimbursement and social health insurance provision for older people and children [14] [15].

To assume the dual responsibilities regarding supplementing suppliers and demanders, China began the fourth reform of its healthcare system by carrying out novel medical sector reform in 2009, with one of the priorities being to improve the healthcare compensation system. The government tripled its financial expenditure on healthcare to alleviate the problems of difficult and expensive access to healthcare [16]. Currently, the government’s financial investment in the healthcare sector is increasing, but the growth rate is decreasing annually, according to data from the China Statistical Yearbook. China is undergoing a reform towards universal health coverage (UHC) but still needs to enhance equality [2] [17]. On the demand side, the government has focused on subsidizing basic healthcare services and undertaking healthcare pricing reforms to improve access to healthcare for all citizens, especially in rural areas [18]. On the supply side, the government has established a tiered diagnosis and treatment system aimed at optimizing the supply of medical services at different levels to achieve a balance between supply and demand and to ensure that medical resources are effectively allocated [19].

The relationship between supply and demand is the basic logic of market operation, and many studies have used this as an entry point to analyse the strategic behaviours and impacts of both supply and demand resulting from government financial compensation [20–22]. Cost-sharing between supply and demand affects the equity of health systems [23], and health system reform can be carried out better if health expenditures are properly arranged [24]. Studies have shown that supply-side cost-control measures have had some success in slowing the rate of cost growth, and demand-side factors, such as the allocation of out-of-pocket costs, affect overall health care spending [25]. It is generally believed that the health of individuals and families can be improved through compensation on the demand side [26]. However, it is debatable whether compensation on the supply side improves the overall quality of the industry, the health of the population and the cost of healthcare [27]. The relationship between healthcare spending and economic performance is complex, and there is ongoing debate about the optimal level of health care spending [28].

Currently, most studies focus on the perspective of a single party, ignoring the coordination between supply and demand. For example, studies have examined how needs can be met through improvements in private health insurance [29] [30], while others have focused on the mode of hospital operation. Hospitals often need to make trade-offs between the quality and cost of healthcare services, and their methods of operation or operational management tools reflect the choice between the two [31]. Government incentives in the form of financial compensation can influence hospitals’ choices between the quality and quantity of healthcare services [32]. Studies have revealed that government subsidies for patient premiums can encourage hospitals to reduce readmission rates and improve the quality of medical services while keeping the quantity unchanged [33], [34]. Studies also focus on the patient perspective. A study from Boston argued that increasing the number of physicians tends to increase supply-side-induced demand [35]. Furthermore, by analysing the relationship between the number of doctors per capita and patient costs, some studies argue that induced demand causes patients to pay more for ineffective care [36]. However, the imbalance between the supply and demand structure is a potential problem in China’s healthcare system, and it is important to provide reasonable financial compensation for both supply and demand [17] [37]. From this point of view, patients will be able to reduce the cost of medical care and medicines, as well as the number of hospitalization days, and hospitals will be able to effectively improve the quality of their medical services [38–40].

Therefore, this study adopts a comprehensive perspective and considers the efficiency of financial subsidies on both the supply and demand sides. This study takes residents’ health and medical expense burden as the entry point and constructs panel data with residents’ health and medical expenses as the explained variables. The data reliability is improved by combining nationwide data from government accounts documents and considering the endogeneity of funds by analysing the hysteresis effect of financial capital investment as a robustness test. Finally, the existence of regional heterogeneity is verified. Through empirical analysis, suggestions for reforming the government’s financial compensation mechanism are proposed.

## Methods

### Data sources

This study analyses the changing trend of financial compensation on the demand and supply sides, residents’ health levels, and medical expenses in 31 provincial-level administrative regions across the country from 2005 to 2019. According to the regional division method adopted in the national data query system of the National Bureau of Statistics, this study divides 31 provinces and cities into three regions: eastern, central, and western. The data used come from public databases, such as the “China Statistical Yearbook,” “China Health Statistical Yearbook,” and “Government Final Account Report”.

### Variables

The explained variables in this study are residents’ health and medical expenses. In discussions of government investment in health care and regional heterogeneity regarding health outcomes and economic growth, perinatal and maternal mortality are the most strongly affected and predominantly discussed health indicators, followed by per capita outpatient and hospitalization costs, which are the most widely used economic indicators [41] [42]. Residents’ health is measured by the perinatal mortality rate and maternal mortality rate; residents’ medical expenses are reflected by per capita outpatient and emergency expenses and per capita hospitalization expenses.

The core explanatory variables are the quotas of the financial “replenishing demand side” and “supplementing supply side” measures of financial compensation. The supplementary demand side indicator reflects financial support from the demand side, such as government support to patients, and is represented by the amount of investment in medical insurance. The supplementary supply side indicator reflects government financial support for healthcare institutions and is expressed by the amount of local financial investment in healthcare institutions. In addition to being affected by economic factors, such as government funding, the financial compensation model, and investment, residents’ health is influenced by sociodemographic factors, such as the population’s education level, living habits, and life concepts [14].

The control variables in this study are divided into two categories. The first category includes indicators related to medical resources, such as the number of beds in medical institutions, the number of doctors, the number of medical technicians, and the proportion of tertiary hospitals. The second category consists of indicators related to population characteristics, including the proportion of illiterate individuals, the urban resident population, and the elderly dependency ratio [43]. Therefore, relevant control variables are selected from these influencing factors according to the studies mentioned above (Table 1).

**Table 1:** Related indicator variables

	Variable name	Indicator application form
Explained variable	Resident health	Perinatal mortality rate by province
		Maternal mortality rate by province
	Resident medical expenses	Logarithm of outpatient and emergency expenses per capita in each province
		Logarithm of the per capita hospitalization expenses in each province
Core explanatory variable	Supplementary funding on demand side	Logarithmic value of provincial government investment in social medical insurance
	Supplementary funding on supply side	Logarithmic value of provincial government investment in healthcare institutions
Control variable	Number of beds	Beds per 1,000 people by province
	Number of health technicians	Number of health technicians per 1,000 people in each province
	Number of doctors	Number of doctors per 1,000 people by province
	Illiteracy ratio	Proportion of illiterate people aged 15 and older in the province
	Old age dependency ratio	Dependency ratio of elderly population by province
	Sex ratio	Male to female population ratio by province
	Permanent residents	Permanent population of cities and provinces
	Proportion of tertiary hospitals	Proportion of tertiary hospitals in each province to all hospitals

### Analytical methods

In this study, residents’ health level and medical expenses are the explained variables, and the supplementary funding on the demand side and supplementary funding on the supply side are the core explanatory variables used to construct a fixed effect model (Eq. 1).

$$\begin{array}{c}{Output}_{it}={\beta }_{0}+{\beta }_{1}{Financial}_{it}+{\beta }_{2}{{\chi }}_{it}+{\alpha }_{it}+{\epsilon }_{it}\left(1\right)\end{array}$$

The explained variables ${Output}_{it}$ are residents’ health level and residents’ medical expenses. The perinatal mortality rate and life expectancy reflect residents’ health level, and the per capita outpatient and emergency expenses and per capita hospitalization expenses are used to reflect residents’ medical expenses. The subscript $i$ refers to the province, and $t$ refers to the year. ${Financial}_{it}$ refers to the financial compensation that province $i$ provides to the supply side and the demand side at time. ${\beta }_{1}$ is the coefficient of interest in this study. Its positive and negative values represent the direction of the impact of government financial compensation on residents’ health and medical expenses, and its absolute value reflects the degree of impact. ${{\chi }}_{it}$ is a series of control variables. The selected control variables include the number of medical technicians, the proportion of tertiary hospitals, the proportion of illiterate individuals, the urban permanent population, and the elderly dependency ratio. ${\alpha }_{it}$ represents the fixed effect at the provincial level, ${\beta }_{0}$ is a constant term, and ${\epsilon }_{it}$ is a random disturbance term.

Next, instrumental variables are introduced to solve the endogeneity problem in regression. Two instrumental variables are constructed: the level of provincial government fiscal revenue and the level of local budgetary expenditure. Regional fiscal revenue and expenditure have a crucial impact on the supply and demand sides and have no direct relationship with hospital preference or patient behaviour, mainly affecting residents’ health and medical expenses. Therefore, financial revenue and expenditure can be instrumental variables in analysing the impact of fiscal supplementary funding and medical expenses (Eq. 2).

$$\begin{array}{c}{y}_{it}={\eta }_{0}+{\eta }_{1}{\text{{\rm Z}}}_{it}+{\eta }_{2}{{\chi }}_{it}+{\alpha }_{it}+{\epsilon }_{it}\left(2\right)\end{array}$$

where ${y}_{it}$ refers to province $i$’s supply and demand quotas at time $t$, and ${\text{{\rm Z}}}_{it}$ is the instrumental variable used in this study. The province fixed effect is also included in the model. ${{\chi }}_{it}$ is a series of control variables, including the number of beds, number of health technicians, proportion of illiterate individuals, elderly dependency ratio, and GDP. Clustered standard errors are controlled at the provincial level. According to the above formula, the predicted value is obtained and then brought into the second regression stage to obtain the corresponding coefficient.

## Results

### Fixed effect regression

This study analyses trends in financial compensation on the demand and supply sides, population health levels and population healthcare costs in 31 provinces across the country over the period 2005–2019 (Table A1-A5). By 2019, the national average maternal mortality rate had decreased by 74.29% and the perinatal mortality rate decreased by 58.94% compared with the values in 2005, with the degree of change varying by region. In addition, government subsidies on both the supply and demand sides, per capita healthcare costs, medical resources in all regions and overall healthcare quality increased annually.

For the demand side, the government’s financial compensation can significantly reduce the maternal mortality rate (Table 2). For every 1% increase in government compensation to the demand side, the maternal mortality rate decreases by 8.461%. Although government demand-side supplementary funding does not significantly impact perinatal mortality, the coefficient is still negative. The fixed effect regression results with per capita outpatient expenses and per capita hospitalization expenses as the dependent variables show that the government supplementary funding on the demand side has a significant positive correlation with per capita outpatient expenses, with a coefficient of 0.280, *p* < 0.001, and the difference is statistically significant. There is also a significant positive correlation between government supplementary funding on the demand side and per capita hospitalization expenses, with a coefficient of 0.204, *p* < 0.001, and the difference is statistically significant. Comparing the coefficients of per capita outpatient and per capita hospitalization expenses, government subsidies on the demand side promote per capita outpatient expenses to a greater extent. The government’s demand-side supplementary funding can significantly improve residents’ health levels and substantially increase residents’ medical expenses.

**Table 2:** Fixed-effect regression of the impact of supplementary funding on the demand side and the supply side on residents’ health and medical expenses

	Supplementary funding on the demand side				Supplementary funding on the supply side			
	Perinatal mortality rate	Maternal mortality rate	Outpatient expenses per capita	Hospitalization expenses per capita	Perinatal mortality rate	Maternal mortality rate	Outpatient expenses per capita	Hospitalization expenses per capita
Supplementary demand/supply side	-1.302	-8.461*	0.28***	0.204***	-1.124***	-2.786	0.112***	0.136***
	(0.836)	(4.762)	(0.043)	(0.039)	(0.229)	(2.630)	(0.019)	(0.017)
Number of beds per 1,000 people	-0.181	0.061	-0.001	0.009	-0.573***	-6.388***	0.071***	0.048***
	(0.3)	(1.709)	(0.015)	(0.014)	(0.143)	(1.646)	(0.012)	(0.011)
Number of health technicians per 1,000 population	-0.391	-3.792*	0.041**	-0.036**	-0.351	2.098	-0.003	-0.032
	(0.367)	(2.088)	(0.019)	(0.017)	(0.283)	(3.250)	(0.023)	(0.021)
Number of doctors per 1,000 population	-101.662	4407.783*	-16.793	41.815**	306.791	-3300.19	-1.43	36.038
	(420.53)	(2395.981)	(21.649)	(19.38)	(376.168)	(4318.478)	(30.918)	(28.514)
Illiteracy ratio	-8.678	-8.3	-0.031	-0.134	15.552***	39.444	-0.161	-0.081
	(9.293)	(52.946)	(0.478)	(0.428)	(4.644)	(53.319)	(0.382)	(0.352)
Old age dependency ratio	1.392	26.916	-0.514**	0.405*	1.261	203.725***	0.089	0.094
	(4.641)	(26.441)	(0.239)	(0.214)	(4.389)	(50.386)	(0.361)	(0.333)
Sex ratio	-0.022	-0.009	0.001	0.002**	-0.046**	-0.35	0.005***	0.005***
	(0.018)	(0.103)	(0.001)	(0.001)	(0.021)	(0.243)	(0.002)	(0.002)
Permanent residents	0.066	0.53	0.008	0.004	0.095***	0.531	-0.003	-0.005*
	(0.052)	(0.297)	(0.003)	(0.002)	(0.034)	(0.395)	(0.003)	(0.003)
Proportion of tertiary hospitals	2.227*	7.135	0.059	-0.077	-2.138	-18.041	0.492***	0.211*
	(1.309)	(7.458)	(0.067)	(0.06)	(1.68)	(19.283)	(0.138)	(0.127)
Intercept	27.591***	111.579*	0.675	5.593***	25.364***	76.235**	3.029***	6.577***
	(10.345)	(58.942)	(0.533)	(0.477)	(3.163)	(36.307)	(0.26)	(0.24)
Observations	117	117	117	117	372	372	372	372
R²	0.5656	0.2609	0.911	0.836	0.709	0.2918	0.776	0.714
F	12.29	3.33	97.146	48.178	89.794	15.2	128.027	92.134

*Note* *, **, and *** indicate significance at the 10%, 5%, and 1% levels, respectively. The numbers in parentheses represent the supply-side coefficients

There is a significant negative correlation between the government’s supplementary funding on the supply side and perinatal mortality; the coefficient is -1.124, *p* < 0.001, and the difference is statistically significant. There is also a negative correlation with maternal mortality, but the coefficient is insignificant. Moreover, government supplementary funding on the supply side has significant positive correlations with per capita outpatient expenses, with a coefficient of 0.112, *p* < 0.001, and per capita hospitalization expenses, with a coefficient of 0.136, *p* < 0.001. Therefore, government supplementary funding on the supply side can significantly improve the health level of residents and increase their medical expenses. However, the coefficients of per capita outpatient and per capita hospitalization expenses are smaller in the supply-side model than in the demand-side model, and the supply-side model has a lower degree of cost promotion.

### Instrumental variables 2SLS regression

Table 3 shows the 2SLS regression results for the impact of the government’s supplementary funding on the demand side on residents’ health and medical care. The direction of the two-stage regression results in the 2SLS regression is the same as that of the fixed-effect regression results. However, the fixed-effect regression overestimates the impact of demand-side government supplementary funding on residents’ health and medical expenses. First, increasing financial compensation to the demand side significantly reduces maternal mortality but has no significant impact on perinatal mortality. The coefficient of the 2SLS regression shows that for every 1% increase in supplementary demand-side fiscal expenditure, the maternal mortality rate decreases by 3.667%. Second, increasing financial compensation to the demand side significantly increases per capita outpatient and per capita hospitalization expenses. The regression coefficient shows that for every 1% increase in compensation to the demand side, per capita outpatient expenses increase by 0.108%, and per capita hospitalization expenses increase by 0.143%. The government’s financial compensation to the demand side thus increases residents’ medical expenses.

**Table 3:** Results of 2SLS regression for the impact of government supplementary funding on residents’ health and medical care

	Supplementary funding on the demand side				Supplementary funding on the supply side			
	Perinatal mortality rate	Maternal mortality rate	Outpatient expenses per capita	Hospitalization expenses per capita	Perinatal mortality rate	Maternal mortality rate	Outpatient expenses per capita	Hospitalization expenses per capita
Supplementary demand/supply side	-0.58	-3.667*	0.108***	0.143*	-1.765**	-5.477	0.225***	0.196***
	(0.673)	(1.879)	(0.021)	(0.074)	(0.765)	(7.823)	(0.066)	(0.073)
Number of beds per 1,000 people	0.701**	3.678***	-0.036*	-0.165***	0.593	0.919	-0.021	-0.099***
	(0.277)	(0.848)	(0.02)	(0.037)	(0.456)	(2.285)	(0.019)	(0.026)
Number of health technicians per 1,000 population	-1.478***	-3.058***	0.047	0.051	-1.061*	-1.425	-0.031	-0.038
	(0.377)	(1.010)	(0.036)	(0.041)	(0.571)	(3.637)	(0.046)	(0.055)
Number of doctors per 1,000 population	1301.567**	3045.728***	34.112	77.412	1106.568	2758.784	115.952	213.275
	(509.241)	(1115.951)	(47.295)	(60.749)	(733.131)	(3687.914)	(54.941)	(62.801)
Illiteracy ratio	6.928	58.197*	-2.9***	-2.828***	17.714***	309.777***	-0.988***	-0.336
	(7.987)	(31.576)	(0.531)	(0.955)	(5.121)	(30.890)	(0.341)	(0.456)
Old age dependency ratio	-26.982***	-82.998***	1.455***	2.088***	-41.462***	-71.707	2.324***	1.112
	(7.187)	(28.818)	(0.546)	(0.708)	(13.741)	(60.285)	(0.617)	(0.716)
Sex ratio	-0.005	-0.073	0.004*	0.012***	-0.074	-0.306	0.005	0.005
	(0.022)	(0.092)	(0.002)	(0.004)	(0.053)	(0.356)	(0.004)	(0.005)
Permanent residents	-0.002	0.015	-0.003***	-0.004**	0.003	-0.018	-0.004***	-0.003**
	(0.014)	(0.039)	(0.001)	(0.002)	(0.013)	(0.108)	(0.001)	(0.001)
Proportion of tertiary hospitals	2.993	-2.705	0.236	0.671***	-1.246	-21.487	-0.135	0.609**
	(2.321)	(4.980)	(0.176)	(0.178)	(5.267)	(25.488)	(0.265)	(0.305)
Intercept	17.239*	71.239***	3.305***	5.827***	41.995***	113.400	1.582*	5.535***
	(8.915)	(18.840)	(0.368)	(0.968)	(9.748)	(25.488)	(0.872)	(0.835)
Observations	117	117	117	117	372	372	372	372
R²	0.6279	0.6223	0.8956	0.9015	0.6606	0.7015	0.7582	0.7446
Wald chi2	81.21	248.23	808.27	516.84	354.77	529.17	512.46	261.89

*Note* *, **, and *** indicate significance at the 10%, 5%, and 1% levels, respectively. The numbers in parentheses represent the coefficients of supply-side supplementary funding

In this study, STATA was used to perform the Hausman test (Table 4), and the results of the test indicated that the illustrative IV regression significantly differed from the original regression, and the results of each regression model were significant at the 0.1 or 0.01 level. Therefore, the fixed effect regression model is selected in this paper.

**Table 4:** Hausman test

Dependent variable	Independent variable	Chi-Sq. Statistic	P
Perinatal mortality	Supplementary funding on demand side	12.12	0.0968
Maternal mortality rate	Supplementary funding on supply side	50.9	0.0000
Maternal mortality rate	Supplementary funding on demand side	23.81	0.0012
Outpatient expenses per capita	Supplementary funding on supply side	79.38	0.0000
Outpatient expenses per capita	Supplementary funding on demand side	34.32	0.0000
Hospitalization expenses per capita	Supplementary funding on demand side	81.64	0.0000

An effective way to address endogeneity is to ensure that the instrumental variables are not weak instruments; therefore, this study conducts a weak instrumental variable test (Table 5). According to the results, the instrumental variables selected for this study all have F values greater than 10, so they do not suffer from the weak instrumental variable problem, which demonstrates the validity of the instrumental variables in this paper.

**Table 5:** Weak instrumental variable test

Variable	Supplementary funding on the demand side	Supplementary funding on the supply side
Robust F	65.129	150.378
P	0.0000	0.0000
Minimum eigenvalue statistic	144.304	375.383

The above tables report the 2SLS regression results of the impact of the government’s financial subsidies on residents’ health and medical expenses. Increasing financial compensation to suppliers significantly reduces perinatal mortality but has no significant effect on maternal mortality. For every 1% increase in the financial supplementary supply, the perinatal mortality rate decreases by 1.765%, indicating that the government’s financial compensation to the supply side can improve the health of residents.

Increasing financial compensation to suppliers significantly increases per capita outpatient and per capita hospitalization expenses. The regression coefficient shows that for every 1% increase in compensation to the demand side, per capita outpatient expenses increase by 0.225%, and per capita hospitalization expenses increase by 0.196%. This result is inconsistent with the theoretical derivation. Research hypothesis 3 is not supported; supplementary funds to providers increase residents’ medical expenses. As scholars who hold the “replenishment and demand side” perspective say, medical institutions can obtain financial compensation from both the government and patients due to their monopoly position.

In addition, the proportion of illiterate individuals and the old dependency ratio significantly affect per capita outpatient expenses. Per capita outpatient expenses decrease by 0.988% for every 1% increase in the illiteracy ratio and increase by 2.324% for every 1% increase in the elderly dependency ratio. Individuals with higher levels of education possess more health knowledge, pay more attention to their health and are more likely to visit outpatient clinics. The greater the elderly dependency ratio is, the greater the proportion of older people. Because the elderly population is more likely to face various diseases, their outpatient expenses are greater, affecting per capita outpatient expenses. Regarding medical resources, the number of beds per 1,000 people will significantly negatively affect per capita hospitalization expenses. For every 1% increase in beds per 1,000 people, per capita hospitalization expenses decrease by 0.099%. According to theoretical deduction, medical institutions have the incentive not to keep beds vacant. When the number of beds increases, medical institutions may hospitalize patients with low disease severity, decreasing per capita hospitalization costs. In summary, increasing financial compensation to providers positively impacts perinatal mortality but leads to higher per capita outpatient and hospital expenditures. The illiteracy rate, the elderly dependency ratio, and the number of beds per 1,000 inhabitants also significantly affect health expenditures.

Given the significant differences in the level of development between regions in China, this study divides the samples into eastern, central, and western subsamples for a regional heterogeneity test. Objectively, the eastern region has the highest economic level, the central region has the second highest economic level, and the western region has the lowest economic level. The level of medical resources and technology also varies significantly between regions depending on their different technological and economic development levels, and government financial inputs may yield different outputs.

**Fig. 1:**
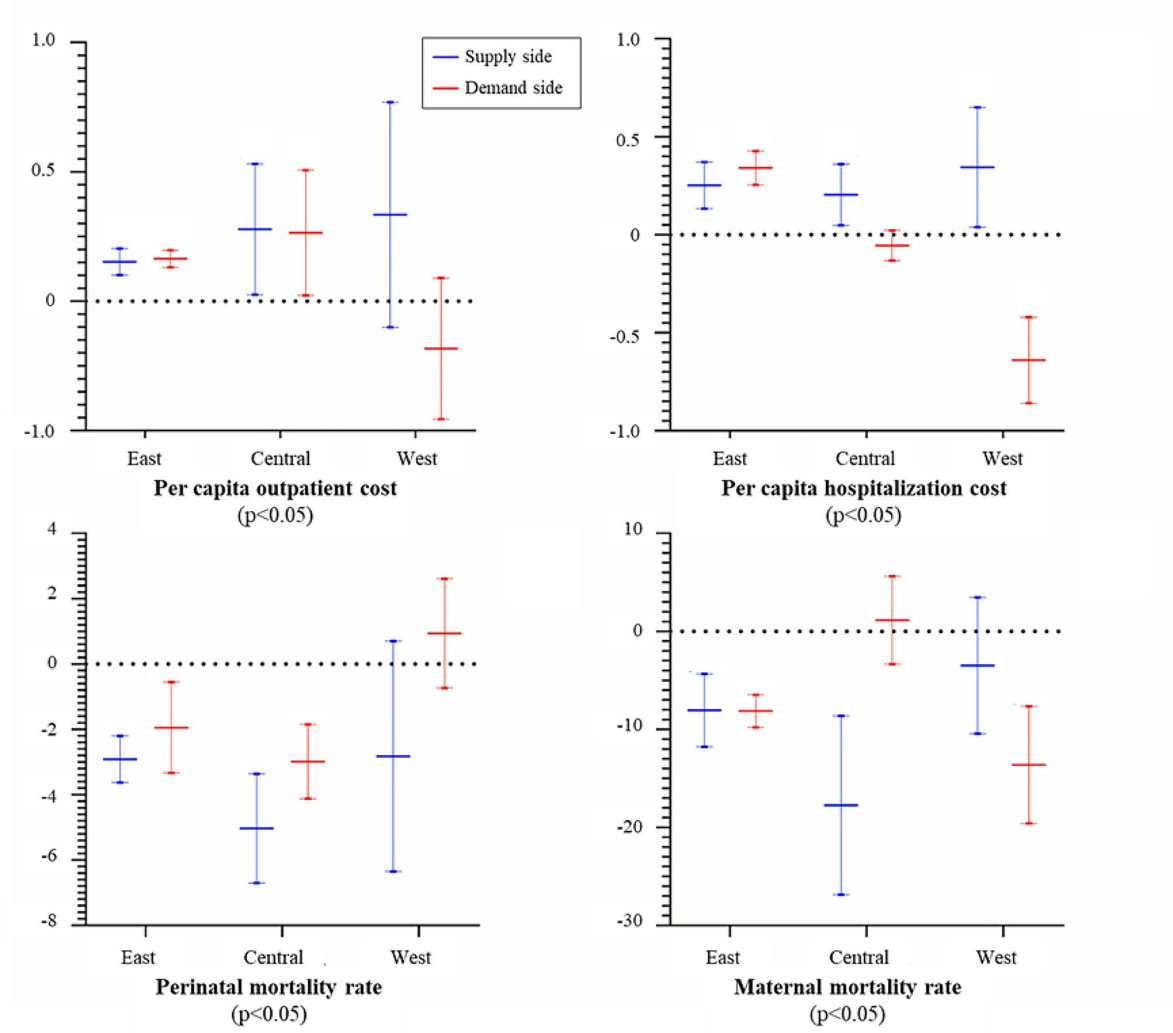
Regional heterogeneity of government

As shown in Fig. 1, there are significant regional differences in the impact of government finance on the health of the population and the cost of medical care on both the supply and demand sides. Whether considering government funding on the supply-side or the demand-side, the effect can significantly improve residents’ health and medical costs in the eastern region. Moreover, the impact of supply-side subsidies on the health level of the population is greater, while the cost-promoting effect is lower. In the central region, supply-side funding has a more significant effect on the population’s health level and the cost of medical care than demand-side funding. Supply-side financial subsidies significantly increase per capita hospitalization costs in the western region. Nevertheless, they have no significant effect on the health level of the population, as represented by the maternal and perinatal mortality rates. On the other hand, fiscal demand-side subsidies can significantly reduce per capita hospitalization costs and improve the health of the population. This is because the western region is less economically developed than the central and eastern regions, and patients’ individual needs are constrained by personal income and the price of medical services. Therefore, via the provision of subsidies on the demand side, patient demand can be reduced, influencing patients’ healthcare behaviours and improving the population’s health.

## Conclusions

China’s method for allocating financial resources in healthcare has gradually shifted from a “one-sided” approach to a “combined demand-supply” financial allocation model. Against the backdrop of a shortage of public expenditure and a gradual slowdown in government investment in healthcare, it is increasingly important to maximize the impact of limited financial resources [44]. Effectively reducing residents’ medical costs is one of the essential goals of China’s deepening healthcare reform, and it is widely recognized in the academic community that government financial compensation can effectively curb the increase in medical costs [45]. Nevertheless, the exact benefits of this healthcare compensation system are not yet clear [46]. Therefore, using provincial panel data from 2005 to 2019, this research explores the impact of residential healthcare benefits and costs when financial compensation is applied on both the supply and demand sides.

The fundamental purpose of government investment in health is to improve the population’s health [47]. This study analyses the impact of government funding on both the supply and demand sides on population health using instrumental variables. Government funding on the supply side can significantly reduce the perinatal mortality rate but has no significant effect on the maternal mortality rate. In addition, governmental subsidies on the demand side significantly reduce the maternal mortality rate but do not substantially affect the perinatal mortality rate.

The findings also suggest that offering compensation on the demand side may further increase healthcare costs instead of controlling healthcare costs. The main reason for this phenomenon is the monopoly of medical institutions and the information asymmetry between the supply and demand sides, which induces patients to spend more on medical consumption. On the other hand, compensating the patient can unleash the patient’s consumption demand and consumption level and increase outpatient and hospitalization costs. Compensation to the supply side may fuel nonprice competition among healthcare providers, and financial subsidies may be used to purchase more high-tech equipment and devices and attract many talented professionals to attract more patients in the industry. This drives up the total healthcare costs, with patients ultimately bearing the burden of these excess healthcare costs.

At this stage, government subsidies have not played an influential role as a means of providing incentives and constraints; instead, the more financial compensation is provided, the greater the medical costs are [48]. This phenomenon results from problems such as imperfections in the healthcare and financial compensation systems, the lack of implementation of various policies, and the differentiation between urban and rural health insurance. In addition, significant regional heterogeneity occurs in the eastern, central and western regions. In the central and eastern regions, where economic levels are higher, supply-side funding is more effective than demand-side funding. In contrast, demand-side funding is more effective in the western region.

The results of this study show that both compensation on the demand side and compensation on the supply side can significantly improve the health level of the population. Moreover, due to the varying levels of economic development and abundance of medical resources among regions, government financial subsidies to the supply side and the demand side have produced different outputs in each region. Therefore, this study suggests that financial resources be allocated between the supply and demand sides according to the actual situation in each region. With a limited level of subsidy, areas with a lower level of economic development should focus on supplementing the demand side to release patients’ demand for healthcare. Regions with a higher level of economic growth should focus on supplementing the supply side to stimulate medical institutions to improve medical technology.

### Electronic supplementary material

Below is the link to the electronic supplementary material.

Supplementary Material 1

## Data Availability

All data in this manuscript are sourced from "China Statistical Yearbook," "China Health Statistical Yearbook," and "Government Final Account Report." All data can be obtained from public channels such as government websites.
