# Supplementary material for: The economic effect of financial compensation in China’s healthcare system: comprehensive insights regarding supply and demand factors
Source: Health Econ Rev. 2024 Mar 16;14:21. doi: 10.1186/s13561-024-00496-5 (PMC10943810; doi:10.1186/s13561-024-00496-5)
Supplement: Supplementary file 1 — Supplementary Material 1 [file 13561_2024_496_MOESM1_ESM.docx]

**Appendix**

Table A1. Statistics of mortality

|  | Maternal mortality rate by region (1/100,000) | | | | Perinatal mortality rate by region (1/1000) | | | |
| --- | --- | --- | --- | --- | --- | --- | --- | --- |
| Year | Total | East | Central | West | Total | East | Central | West |
| 2005 | 49.55 | 19.1 | 33.82 | 92.86 | 10.96 | 8.08 | 9.83 | 14.77 |
| 2006 | 43.35 | 16.97 | 32.93 | 78.24 | 10.59 | 7.71 | 9.23 | 14.57 |
| 2007 | 39.87 | 16 | 28.19 | 73.31 | 9.68 | 7.25 | 8.6 | 13.01 |
| 2008 | 32.11 | 12.99 | 23.76 | 58.07 | 9.69 | 7.07 | 8.75 | 13.08 |
| 2009 | 28.84 | 12.03 | 20.71 | 52.29 | 8.69 | 6.64 | 7.74 | 11.51 |
| 2010 | 28.84 | 12.03 | 20.71 | 52.29 | 8.69 | 6.64 | 7.74 | 11.51 |
| 2011 | 22.15 | 8.82 | 14.94 | 41.37 | 7.49 | 5.84 | 6.65 | 9.83 |
| 2012 | 20.66 | 8.98 | 14.14 | 37.67 | 7.06 | 5.42 | 6.16 | 9.44 |
| 2013 | 19.78 | 9.36 | 13.92 | 35 | 6.46 | 4.94 | 5.88 | 8.46 |
| 2014 | 17.72 | 8.37 | 14.47 | 29.74 | 6.16 | 4.86 | 5.5 | 7.98 |
| 2015 | 16.31 | 7.29 | 13.3 | 27.81 | 5.78 | 4.34 | 5.13 | 7.76 |
| 2016 | 16.63 | 8.62 | 12.42 | 28.08 | 5.91 | 4.58 | 5.21 | 7.82 |
| 2017 | 16.32 | 9.17 | 13 | 26.18 | 5.39 | 4.04 | 4.65 | 7.33 |
| 2018 | 13.88 | 8.28 | 12.51 | 20.6 | 4.95 | 3.83 | 4.35 | 6.57 |
| 2019 | 12.74 | 7.18 | 11.18 | 19.58 | 4.5 | 3.66 | 4.02 | 5.73 |

Table A2. Input tables of demand side (10 thousand yuan)

| Year | Total | East | Central | West |
| --- | --- | --- | --- | --- |
| 2014 | 4929439 | 5849393 | 3003612 | 3175662 |
| 2015 | 5229628 | 6036403 | 3415196 | 4119906 |
| 2016 | 5803489 | 7029684 | 4617481 | 4527060 |
| 2017 | 5846800 | 7404116 | 4895316 | 4202759 |
| 2018 | 6358616 | 8456459 | 5656559 | 3963461 |
| 2019 | 7021986 | 9335219 | 6260437 | 4370122 |

Table A3. Input tables of supply side (10 thousand yuan)

| Year | Total | East | Central | West |
| --- | --- | --- | --- | --- |
| 2007 | 378469.2 | 420853.8 | 450356.4 | 277267.8 |
| 2008 | 329078.1 | 474143.8 | 275643.9 | 227731.3 |
| 2009 | 430754.2 | 597633.2 | 385901.8 | 300572.6 |
| 2010 | 538024.1 | 726780.6 | 480664.4 | 396198.2 |
| 2011 | 737419.3 | 981118.2 | 676348 | 543687.8 |
| 2012 | 1113398 | 1190922 | 791068.6 | 699598 |
| 2013 | 1010014 | 1362429 | 910811.4 | 738765.1 |
| 2014 | 1128912 | 1516005 | 1002427 | 845308.6 |
| 2015 | 1393970 | 1840256 | 1258429 | 1058582 |
| 2016 | 1564057 | 2098612 | 1399781 | 1163909 |
| 2017 | 1752339 | 2399847 | 1540118 | 1278467 |
| 2018 | 1956406 | 2732639 | 1679500 | 1406731 |
| 2019 | 2206734 | 3086333 | 1927014 | 1555997 |

Table A4. Resident medical expenses (yuan)

|  | Per capita hospital expenses in each region | | | | Per capita outpatient expenditure by region | | | |
| --- | --- | --- | --- | --- | --- | --- | --- | --- |
| Year | Total | East | Central | West | Total | East | Central | West |
| 2005 | 6589.76 | 9260.8 | 5308.63 | 4966.91 | 167.56 | 215.7 | 157.33 | 127.78 |
| 2006 | 6523.76 | 9088.69 | 5403.04 | 4875.79 | 168.96 | 212.61 | 161.31 | 131.56 |
| 2007 | 6705.06 | 9277.38 | 5674.57 | 4975.88 | 171.28 | 214.14 | 165.19 | 133.4 |
| 2008 | 7021.11 | 9618.94 | 5929.23 | 5292.09 | 176.82 | 220.3 | 168.47 | 140.17 |
| 2009 | 7767.92 | 10613.72 | 6621.96 | 5859.74 | 194.21 | 240.22 | 185.46 | 155.35 |
| 2010 | 7882.69 | 10618.37 | 6866.88 | 5978.12 | 194.95 | 238.41 | 186.95 | 158.03 |
| 2011 | 8039.44 | 10734.02 | 7103.86 | 6112.34 | 200.9 | 241.33 | 195.55 | 164.85 |
| 2012 | 8308.48 | 10977.65 | 7480.25 | 6316.94 | 210.91 | 249.39 | 207.99 | 174.83 |
| 2013 | 8709.17 | 11365.45 | 7797.58 | 6798.74 | 223.04 | 258.85 | 217.69 | 191.61 |
| 2014 | 8896.66 | 11648.3 | 8029.81 | 6854.25 | 233.31 | 267.74 | 229.41 | 202.07 |
| 2015 | 9291.9 | 12261.08 | 8265.72 | 7162.32 | 245.57 | 281.99 | 239.32 | 214.26 |
| 2016 | 9515.97 | 12497.41 | 8399.49 | 7448.03 | 252.95 | 291.19 | 244.7 | 221.47 |
| 2017 | 9669.43 | 12794.62 | 8551.67 | 7458.77 | 261.78 | 303.94 | 251.37 | 228.15 |
| 2018 | 9905.13 | 13020.7 | 8802.91 | 7691.37 | 272.49 | 319.62 | 259.66 | 235.86 |
| 2019 | 10188.45 | 13318.76 | 9068.2 | 7974.7 | 283.12 | 329.84 | 271.6 | 245.84 |

Table A5. Descriptive statistics of variables

| Variable | Maximum | Minimum | Mean | Standard deviation | Sample |
| --- | --- | --- | --- | --- | --- |
| Supplementary demand side | 16537432 | 664127.6 | 5692779 | 3684167 | 117 |
| Supplementary supply side | 9468038.38 | 79986.45 | 1227382 | 1080886 | 403 |
| Number of beds per 1,000 population | 7.55 | 1.51 | 4.36 | 1.44 | 465 |
| Number of health technicians per thousand population | 15.46 | 2.1 | 5.36 | 1.95 | 465 |
| Number of doctors per thousand population | 11.35 | 1.01 | 3.7 | 1.53 | 465 |
| Illiteracy ratio | 0.46 | 0.01 | 0.07 | 0.07 | 434 |
| Old age dependency ratio | 0.24 | 0.07 | 0.13 | 0.03 | 434 |
| Sex ratio | 123.17 | 94.65 | 104.22 | 3.94 | 434 |
| GDP per capita | 164563 | 5218 | 40085.06 | 26545.03 | 465 |
